# Episodic Memories and Their Relevance for Psychoactive Drug Use and Addiction

**DOI:** 10.3389/fnbeh.2013.00034

**Published:** 2013-05-23

**Authors:** Christian P. Müller

**Affiliations:** ^1^Section of Addiction Medicine, Department of Psychiatry and Psychotherapy, Friedrich-Alexander-University of Erlangen-NurembergErlangen, Germany

**Keywords:** episodic drug memory, experimental consumption, drug instrumentalization, addiction

## Abstract

The majority of adult people in western societies regularly consume psychoactive drugs. While this consumption is integrated in everyday life activities and controlled in most consumers, it may escalate and result in drug addiction. Non-addicted drug use requires the systematic establishment of highly organized behaviors, such as drug-seeking and -taking. While a significant role for classical and instrumental learning processes is well established in drug use and abuse, declarative drug memories have largely been neglected in research. Episodic memories are an important part of the declarative memories. Here a role of *episodic drug memories* in the establishment of non-addicted drug use and its transition to addiction is suggested. In relation to psychoactive drug consumption, episodic drug memories are formed when a person prepares for consumption, when the drug is consumed and, most important, when acute effects, withdrawal, craving, and relapse are experienced. Episodic drug memories are one-trial memories with emotional components that can be much stronger than “normal” episodic memories. Their establishment coincides with drug-induced neuronal activation and plasticity. These memories may be highly extinction resistant and influence psychoactive drug consumption, in particular during initial establishment and at the transition to “drug instrumentalization.” In that, understanding how addictive drugs interact with episodic memory circuits in the brain may provide crucial information for how drug use and addiction are established.

## Introduction

“I smoked my first cigarette when I was 12 years old – behind the house, together with a few friends and some older guys who had provided it for trying. It was incredibly cool, not allowed of course, and it tasted awful. Despite persistent peer pressure, the memory of this one event still accompanies me until present day. It forms part of my personality and prevents me from trying cigarettes again. My first experience with alcohol a little later at the retirement party of my much beloved grand dad was quite bad as well. I felt sick and abstained from any countable consumption of alcohol for a few years. However, when I was a student I felt forced to social drinking which I tentatively did. At one occasion, however, I learned from rather unexpected feedback that my constitutively restricted social skills were tremendously enhanced after a few glasses of wine. This one episode, again vividly remembered to the present day, has shaped my drug consumption behaviour into another direction. After establishing a working dose window, I can now “instrumentalize” a controlled consumption for just this one purpose: “socializing on demand.”

(*personal communication*)

These admittedly very personal example may illustrate that the availability of a psychoactive drug may lead to very different behavioral patterns in the long run after having established episodic memory of the first (few) drug consumption episodes. But do episodic memories just orchestrate other, more important learning processes at consciously accessible level or do they play a causal role in the establishment of regular drug consumption and addiction?

Psychoactive drugs are chemical compounds which influence our subjective perception and/or our behavior. Human beings consume psychoactive drugs voluntarily (Abel, 1980; Waldorf et al., 1991; Heath, 2000; Amendt, 2003). The consumption may range from a one-trial experience, after which the consummatory behavior is never repeated up to a stage of drug addiction when virtually all behavioral activity is directed toward the consumption of one or more psychoactive drugs. What starts with a single episode, remembered as an episodic memory, may finally control the complete behavioral repertoire of an individual.

Drug addiction is a now regarded a major psychiatric disorder which carries a considerable burden for individuals and their social environment (American Psychiatric Association, 1994). Much like other psychiatric disorders it compromises life quality of affected individuals and their families and has profound economic consequences. There is still no effective treatment available which may reverse the rather complex set of behaviors regarded as addiction, to a condition of either controlled drug use or abstinence. Understanding how drug addiction develops is, therefore, a major quest for behavioral neuroscience and psychiatry. While early views on addiction development were based on the action of psychoactive drugs as pharmacological reinforcers (Koob, 1992; Wise, 1994, 2002; Koob et al., 1998) and addiction as an aberrant learning mediated by the reinforcement system (Di Chiara, 1995), more recent views have acknowledged the role of various memory systems in the establishment of drug use and addiction (Nestler, 2002; Kelley, 2004; Hyman et al., 2006). A recent analysis suggests that virtually all memory systems that humans are capable of might contribute to addiction development (Müller and Schumann, 2011a).

Importantly, not every individual who tries a psychoactive drug and establishes an episodic memory of the subsequent consummatory occasion with its aftermath becomes addicted to the drug. Inter-individual differences based on genetic (Stacey et al., 2009, 2012; Schumann et al., 2011; Easton et al., 2013a) and developmental factors (Campbell et al., 2009; Dong et al., 2011), accentuated personality traits (Piazza et al., 1989; Belin et al., 2008), or comorbid psychiatric disorders (Robbins and Everitt, 1999) appear to predispose individuals differently to the risk of fast establishment of drug consumption and/or the transition from controlled to compulsive drug use. However, most regular consumers of psychoactive drugs do never become addicts. Large consumer surveys in the US (SAMHSA, 2011) and Europe (EMCDDA, 2012) reveal that the majority of the regular consumers of legal drugs like alcohol or illicit drugs like cannabis or cocaine may exert a lifelong control over their consumption (Ahmed, 2010; Müller and Schumann, 2011a). However, a significant minority of those humans and animals, who have established drug-seeking and consumption behaviors lose control and develop a compulsive pattern of consumption (Gawin, 1991; Deroche-Gamonet et al., 2004; Vanderschuren and Everitt, 2004).

While it is currently under debate whether the controlled use of psychoactive drugs might under certain circumstances have beneficial effects for behavioral performance, the achievement of life goals, or wellbeing (Lende and Smith, 2002; Lende et al., 2007; Hagen et al., 2009; Müller and Schumann, 2011a,b), it has to be acknowledged that the behavior of psychoactive drug consumption is established by the majority of humans in the western world and forms a rather stable trait. Since neither humans nor drug consuming animals are developmentally determined to automatically establish drug consumption, it may be assumed that the stability of this trait over generations is based on the capability to learn it by social learning or *de novo* (Müller and Schumann, 2011a). The ability to modify food consumption according to non-nutritional needs may be seen as the phylogenetic origin of psychoactive drug consumption. This capability is already present in various animal species (Rodriguez and Wrangham, 1993; Lozano, 1998; Huffman, 2003). The learning of consummatory behaviors may then involve either a learning by trial-and-error (e.g., for newly emerging substances; Hassan et al., 2013), or by cultural inheritance/learning (Dean et al., 2012). Importantly, any way to acquire drug consumption related behaviors involves learning and memory processes. The high persistence of the behavior after long periods of abstinence suggests high stability and extinction resistance of related memories.

The goal of this paper was to review the role of episodic memories in non-addicted psychoactive drug use and in drug addiction. While there is no such role acknowledged yet, this is to my knowledge the first attempt to make a case for episodic drug memories and their function. I will first consider the role of learning and memories in the establishment of drug use behaviors and review the history of drug- and addiction memory concepts. Then a recently suggested concept of drug memories is discussed which includes for the first time also episodic drug memories. An attempt is made to further refine a potential definition of episodic drug memories. It is discussed when in the time course of establishing drug use and abuse behaviors, they might play a role. Finally I review some of the neuropharmacological evidence for brain processes, potentially related to episodic drug memories. This discussion is meant to introduce episodic drug memories into the frame of relevant memory systems for drug use and addiction. As such it may not be complete and cannot deliver final conclusions, but should be understood as a first suggestion for subsequent debate.

## Drug Use Requires Memories

According to current diagnostic manuals, drug addiction is diagnosed by the incidence of a number of rather complex behaviors which involve, e.g., mental occupation with the drug, active seeking of the drug, and active drug consumption. Most important, it also involves compulsive drug use and relapse long after the drug has left the organism and even long after compensatory processes in the brain have subsided. Thus, it was suggested that drug addiction may be based on memory formation and voluntary and involuntary retrieval.

Although still poorly defined in psychological terms, the concept of a *drug-* or *addiction memory* is not new (Boening, 2001). Mello (1972) introduced the term “memory of addiction” in a discussion of addiction-related behaviors. However, the dominating concept of drug action at that time and later on assumed that the reinforcing effects of psychoactive drugs were mostly dissociated from their interaction with memory systems (White and Milner, 1992). Nevertheless, several lines of evidence suggested important interactions of psychoactive drugs with memory systems. It was shown in animals and humans that drug-associated cues can work as a classically conditioned stimulus and induce either drug-related effects or withdrawal symptoms (Wikler and Pescor, 1967; Siegel, 1975; O’Brien et al., 1977; Siegel et al., 1982). The conditioned withdrawal symptoms, which can be ameliorated almost immediately by new drug consumption, were assumed to contribute significantly to the continuation of the drug use (Siegel, 1988; O’Brien et al., 1998; but, see Drummond et al., 1990). Another line of evidence showed that psychoactive drugs applied post trial can also enhance memory for non-drug-related behaviors (Huston et al., 1974, 1977), thus indicating that drugs do not just shape memories to acute drug-effects, but also of preceding events and behaviors. White (1996), when summarizing the evidence, suggested that the reinforcing effects of addictive drugs may at least in part be brought about by their interaction with multiple memory systems of the brain. He suggested three general types of memory that are influenced independently by psychoactive drugs. These systems would be involved in conditioned incentive learning, declarative learning, and habit or stimulus-response learning. Conditioned incentive learning described the learning of stimulus-incentive associations, after which a neutral cue would become a conditioned reward that is able to elicit conditioned approach behavior (Squire et al., 1993; Milner et al., 1998). Major brain structures mediating the influence of psychoactive drugs on conditioned incentive learning are the amygdala, the nucleus accumbens, and the tegmental pedunculopontine nucleus (White, 1996). Declarative learning describes the learning of relationships among cues which can be neutral in their consequences, also known as stimulus–stimulus learning (Squire et al., 1993; Milner et al., 1998). In contrast to non-declarative memories, the content of declarative memories should be consciously accessible (Squire et al., 1993; Milner et al., 1998). In terms of addiction memory, it contains information on the relationships among external cues and events relevant for drug taking (White, 1996). Brain structures that are involved are the hippocampus network and the neocortex (Milner et al., 1998; Bast, 2007). Habit learning described the learning of stimulus-response associations, which are strengthened by the occurrence of reinforcement. The neural correlate for habit learning is the caudate-putamen (Knowlton et al., 1996; Milner et al., 1998). An important role of habit learning for drug addiction was early recognized in particular for drug self-administration behavior (White, 1989, 1996). This view received important support from more recent studies demonstrating not only the neuroanatomical preconditions (Haber et al., 2000), but also their functional relevance for a transfer of information between stimulus-outcome learning and stimulus-response learning systems (Porrino et al., 2004; Belin and Everitt, 2008). White (1996) argued that the addictive properties of drugs have multiple causes based on their multiple independent interactions with those memory systems which store different aspects of the drug experience and of drug taking behaviors. Another classification of addiction memories was proposed by Heyne et al. (2000). They suggested distinguishing at least three different memory types in relation to drug consumption: a memory of drug-effects, a memory of drug use, and a memory of addiction (Heyne et al., 2000; Boening, 2001).

A more recent view considered the hypothesis that drug addiction may be understood in terms of recruitment of neural systems that normally mediate learning and memory (Robbins et al., 2008). Thereby, drugs are assumed to always work as unconditioned reinforcers which support emotional learning, encompassing Pavlovian as well as instrumental conditioning. The amygdala, nucleus accumbens, and orbitofrontal cortex play important roles in the acquisition and retrieval of emotional memories related to the drug (Kilts et al., 2001; Everitt et al., 2007). For the procedural (habit) learning system, a cascading loop transfer of cue-controlled drug-related behavior from the ventral to the dorsal striatum was suggested (Robbins et al., 2008). It was concluded that “addiction is a product of aberrant associative learning” which might also involve other pathological changes in behavior. A significant part of its aberrant nature might be its compulsive impact on drug-seeking and -taking behavior, which was suggested to be mediated by a loss of prefrontal cortex control over drug-related habits in the dorsal striatum (Belin et al., 2009). Although this model acknowledges important roles of memory circuits in drug addiction, the view on potential types of memories involved in drug use remains somewhat incomplete and limited to the concept of addiction. It should be noted that there appeared a conceptual gap between the concepts of addiction and the supportive literature on brain mechanisms. Virtually all evidence derived from the host of animal studies did actually not measure the syndrome of addiction, but only single drug-associated behaviors which were not established up to the level of compulsiveness (but, see Deroche-Gamonet et al., 2004; Vanderschuren and Everitt, 2004). In that, conclusions on brain mechanisms might predominantly apply to non-addicted drug use and only serve as a starting point for neuronal adaptations related to addiction.

To allow for a more focused research in the subtypes of a drug memory, a nomenclature for different subtypes of memories was recently suggested by Müller and Schumann (2011a). Research in the neuronal mechanisms of non-drug memories has shown that there are different memories with distinct neuronal mechanism (Eichenbaum, 1997; Milner et al., 1998; McGaugh, 2000). It was suggested that a similar differentiation might also help to segregate single types of drug-related memories and elucidate crucial neuronal mechanism. Accordingly, the drug memory nomenclature was expanded to the types of memories originally identified for non-drug-related experiences (Squire et al., 1993; Milner et al., 1998).

**Figure 1:**
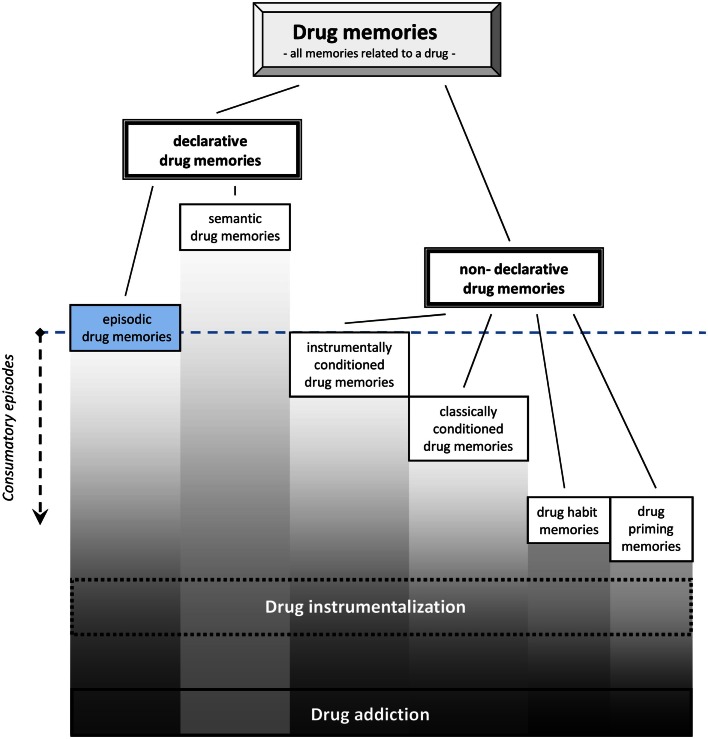
**Episodic and other drug memory systems as they are established during drug consumption**. Note that semantic drug memories may already establish before first drug consumption, while episodic drug memories require only single consumption episode [modified from Müller and Schumann (2011a)].

For drug memory it is suggested to distinguish two major categories: a *declarative drug memory* and a *non-declarative drug memory* (Figure 1). The declarative drug memory contains information that is consciously accessible, i.e., it can be reported verbally by humans. The declarative drug memory should comprise a *semantic memory* for drug facts and one for *drug episodes*. The semantic memory for drugs contains all impersonal facts, rules, and concepts involving drugs, e.g., their names, where they come from, recommended doses, what others report about its effects, and what the rules of their consumption are (Müller and Schumann, 2011a). The establishment of this type of drug memory usually starts before a person is engaged in the first episode of consumption by learning facts from others about the drug (Miller et al., 1990; Leigh and Stacy, 2004). By that way an early semantic drug memory shapes the first expectations of drug-effects, which is then constantly adapted after actual consumption started (Kidorf et al., 1995). It was suggested to conceptualize the expectation of drug-effects (Leigh, 1989; Del Boca et al., 2002) as a retrieval process from different types of memories (Goldman et al., 1991). Also in experienced users, it was shown that the expectation of the drug-effects can still shape the physiological effects of the drug as well as its subjective perception (Volkow et al., 2003, 2006), and thus, influence the establishment of episodic drug memories.

The *episodic drug memory* comprises the memories of all personally experienced episodes with the drug. It is an autobiographical memory of the “What,” “Where,” and “When” of the personal drug encounters. It involves an “autonoetic awareness” of single drug experiences in the continuity of ones subjectively apprehended time. It involves a remembering of the drug episodes as well as “thinking about” the drug when planning for future behavior (Tulving, 2001; Dere et al., 2006, 2008). This may include automatically formed memories of subjectively experienced acute drug-effects, e.g., the mental states the drug induced. The episodic drug memory also comprises aversive withdrawal episodes and periods during subsequent abstinence. These episodes are either characterized by the feeling of drug craving or mental preoccupation with the drug. Clearly, relapse episodes also have episodic memory components. The episodic drug memory system can also contain memories of what was done during a particular drug-induced mental state, and even what effects it had in terms of the environmental feedback (Boening, 2001). In accordance with Tulving’s definition (Tulving, 2002), also episodic drug memories involve the ability for “mental time travel” and “conscious recollection.”

While the experience of “euphoria” or a “high” is often the sought after mental state, it is not the only one occurring on the time scale of a drug consumption episode. A single drug episode may better be considered as a sequence of several distinct mental states. This can be measured as the discriminative stimulus properties of a drug. The discriminative stimulus properties are directly reflecting the mental state induced by a psychoactive drug (Overton, 1968; Stolerman, 1992). As such, this type of memory appears to be crucial for “drug instrumentalization” (Müller and Schumann, 2011a,b). An episodic drug memory requires only a single learning trial, but may be extinction resistant up to a whole life span. Its retrieval may trigger psychoactive drug-seeking and taking, but also passive avoidance of the drug (Eissenberg and Balster, 2000; Miller, 2001).

The *non-declarative drug memory*, in contrast, is not consciously accessible and can only be inferred from behavioral changes in animals and humans. The non-declarative drug memories contain engrams of the classically conditioned drug memory, instrumentally conditioned drug memory, habit memory, procedural drug memories, and drug priming memories.

*Classically conditioned drug memories* may contain all drug-effects that refer to the process of Pavlovian conditioning (Bouton and Moody, 2004). These may include, e.g., the sensitization of the acute drug-effects (Kalivas et al., 1993; Vanderschuren and Kalivas, 2000), drug tolerance, conditioned locomotor activity, conditioned emotional and physiological responses (Foltin and Haney, 2000), conditioned place preference (Bardo and Bevins, 2000; Tzschentke, 2007), and conditioned withdrawal effects (Goldberg, 1975; Siegel, 1988; O’Brien et al., 1998).

*Instrumentally conditioned drug memories* comprise engrams established by instrumental conditioning. Major behaviors induced by the retrieval of those engrams are drug-seeking behaviors and drug self-administration (Spealman and Goldberg, 1978; Richardson and Roberts, 1996). These memories also include drug-cues which are rewarding by themself, as shown in conditioned place preference (Huston et al., 2013), or which can re-instate drug-seeking and drug self-administration behavior (de Wit and Stewart, 1981; Shaham et al., 2003).

*Drug habit memories* refer to instrumental behavior that is no longer goal directed, but stimulus controlled, i.e., a behavioral response that is triggered by a cue, but independent from its behavioral consequences (Everitt and Robbins, 2005). This type of memory is not only important for the transition from controlled to compulsive drug use and addiction (Porrino et al., 2004; Belin and Everitt, 2008), but may already in non-addicted drug users play a role in stimulus driven drug instrumentalization.

*Procedural drug memories* comprise all memories for skills involved in handling a drug. This may range from its production (e.g., cooking up heroin; rolling a joint with marijuana) to the actual way of self-administration (e.g., snorting cocaine; setting a needle for an i.v. heroin injection).

The *Drug priming memories* refer to those engrams whose activation by a small amounts of the drug, which would not induce major subjective and behavioral effects in drug naïve individuals, may in an experienced user induce drug-related behavior (e.g., re-instate drug-seeking, conditioned place preference, or self-administration) and subjective effects (Figure 1).

It was recently suggested that the majority of non-addicted humans, who consume psychoactive drugs as an integral part of their lives (O’Malley and Johnston, 2002; Skogen et al., 2009) take drugs because the subsequent effects can be used for their personal goals in a systematic way. Evidence reviewed by Müller and Schumann (2011a,b) suggests that psychoactive drugs can be “*instrumentalized*,” i.e., used like an instrument. Thereby, “*drug* *instrumentalization*” was defined as a two-step behavioral complex of two interconnected processes: (a) the seeking and consumption of a psychoactive drug in order to change the present mental state into a previously learned mental state, which then allows for (b) a better performance of other, previously established behaviors and a better goal achievement by these behaviors. It was suggested that drug instrumentalization requires the interplay of various, if not all, types of drug memories which need to be established during different stages of experimental consumption (Müller and Schumann, 2011a).

It was suggested that drug-related behaviors, including self-administration, can be learned and maintained in several ways, which may also involve contributions of several memory systems to what appears as one behavioral output, but may in fact contain many different sequences (White, 1996). Externally induced or spontaneous retrieval from certain types of the drug memories allows for a systematic instrumentalization of the drug’s psychotropic effects. Thereby, episodic and instrumentally conditioned drug memories may be considered as the most important memories for drug instrumentalization.

It should be noted that the occurrence of these memories does not automatically implicate a transition to addition. In fact, most people with drug experience are able to manage drug use and instrumentalization in order to gain a fitness benefit within socially approved limits during their entire life. A drug memory is, therefore, not an indicator for drug addiction. Learning and memory research in relation to psychoactive drugs aimed mostly at defining the memory in relation to addiction as an *addiction memory*, although animal models depicted mostly non-addicted consumption; Olmstead, 2006; Sanchis-Segura and Spanagel, 2006). In line with White (1996) and Heyne et al. (2000), a distinction between *drug memory* and *addiction memory* is suggested, based on the degree of elaboration and the compulsive nature that the retrieval of memories has in addiction (Figure 1). In that, the drug memory should comprise all information about psychoactive drugs as defined above, whereas an addiction memory contains quantitatively more of this information with stronger engrams that are more powerful to suppress non-drug memories. Considering the compulsive nature of drug addiction, it cannot be ruled out that there will also be some sort of qualitative differences in the engrams and/or the retrieval of the memories.

## The Specific Nature of Episodic Drug Memories

Episodic memories are major constituents of an individual personality. The encoding and later retrieval of episodes and experiences shapes individual biographies and makes memories accessible for individuals later in live. This also comprises the psychoactive drug biography, i.e., the consumer history of an individual. In that single consummatory episodes may be the substrate of what is remembered in an episodic drug memory. In contrast to non-drug episodes, there are some specific properties to the “What,” “Where,” and “When” criteria of episodic drug memories. The classical concept of episodic memory refers to three external dimensions; the “What” refers to an event or series of external stimuli, the “Where” is an external place, and the “When” describes subjectively anchored time referenced to an individual’s life (Tulving, 2001). Episodic drug memories may expand all three components by a subjective and social cognitive component:

*What*: the “*What*” may be best defined as the drug-effect on the mental state of an individual, in fact being a series of introspectively perceived mental state changes. This usually happens parallel to perceived changes in autonomous system activity (e.g., sickness after a heroin injection) and is influenced by external events (e.g., dance music during an ecstasy episode). One drug episode may involve several different mental states. As, for example, an alcohol episode may involve the sequence of “sobriety – slight disinhibition – emotional high – sedation – hangover with headaches.”
*Where*: drug-effects do not only depend on the dose of a particular substance but also on the *set* and *setting* of the individual (Zinberg, 1984). As such the “Where” component may comprise not only a spatial environment, but the overall setting of a consummatory episode. Thereby the *setting* involves social parameters such as peers present and psychological factors like peer group pressure. The *setting* also involves expectations on the subjective effects of the drug that is about to be consumed, and on emotional arousal derived there from Volkow et al. (2003, 2006).
*When*: the “When” refers to a temporal localization of an event in the individuals own biography. However, this may also include the *set* of a person at this time point, i.e., the physical constitution and mental state a person was in at the moment of drug-seeking and consumption.

The earliest episodic drug memories of an individual are established upon the first encounter with a drug when the experimental consumption has no other goal than to experience the mental state change and other physiological effects induced by a psychoactive drug. In its simplest form, episodic drug memories may be a compound percept of a time in personal history with a particular mental state (When), a spatial location with all present social influences (Where), and the mental (and physiological) state change that the drug induced (What). This may for instance be the memory of an episode when a slightly anxious and aroused adolescent person tries alcohol for the first time in a group of friends following social pressure to do so and experiencing a burning taste with some dizziness afterward.

Once drug consumption has been initially established, but is not at the level of a habit yet, there is another important occasion when episodic drug memories come into play: the establishment of “drug instrumentalization.” It was argued before that during experimental consumption, humans and animals do not only learn the set and setting of drug-effects, but also the instrumentalization of a drug (Müller and Schumann, 2011b). Thus, it is postulated that a particular type of episodic drug memories will include additional components related to the drug’s instrumentalization, such as:

*What*: the beneficial effects on non-drug-related behavior by a drug-induced change of mental state.
*Where*: a place or situation where both are available/possible, a particular psychoactive drug and a chance to perform a goal-directed instrumental behavior.
*When*: a given situation, where the present mental state of an individual appears to be a suboptimal state for a desired goal-directed behavior, so that a mental state change is desirable.

The components of a drug-instrumentalization episodic memory may for example include engrams of a pub with alcohol and potential sex partners available as chance for rewarded behavior (Where). It may include the memory of a date and time but also of a stressed and tired mental state with the wish to change this to a relaxed and slightly disinhibited mental state (When). Finally it may comprise the memory of having a few drinks that changed the initial to the desired mental state with a subsequent successful social interaction and securing a partner for life (What).

Evidence for these types of compound drug memories may be derived from numerous verbal reports of drug users that are able to consciously retrieve rather complex “What-Where-When” information of their drug-instrumentalization episodes (Waldorf et al., 1991; Heath, 2000; Boys et al., 2001; Boys and Marsden, 2003; Boyd et al., 2006; Frederiksen et al., 2012). One may argue that these are conscious self-analyses of one’s own instrumentally conditioned behavior. However, it is well known that instrumental conditioning, in particular of complex behavioral sequences, requires numerous learning trials. Many drug users, however, can report single events as crucial and sometimes life-changing episodes. This might favor the view that there are persistent one-trial drug memories which are very complex and extinction resistant.

While there is no doubt on whether there are episodic drug memories, it is not entirely clear which causal role they might play in the course of establishing drug consumption, instrumentalization and, possibly, addiction. Here it is suggested that episodic drug memories play important roles at least at two different stages in the etiology of drug use: during the initiation of consumption and at the transition to drug instrumentalization.

Drug use behaviors start with experimental consumption usually during adolescence (Sher et al., 2005) with a rather undifferentiated consumption (Spear, 2000; Kuntsche et al., 2005). Experimental consumption, in contrast to instrumentalization and compulsive consumption, refers to a consummatory behavior of which the consequences are initially unknown to the individual. The introduction to the drug in appropriate settings is usually done by older and/or more experienced members of the peer group (e.g., Friedman et al., 1985; Eissenberg and Balster, 2000). However, given inter-individual differences in drug pharmacokinetics and -dynamics, in personality, and in life circumstances, each person has to customize the drug use. It should be noted that although there are expectancies of the drug-effects in drug naïve consumers (Miller et al., 1990), the individual response profile after first consumption is often unpredictable (e.g., Waskow et al., 1970; Jones, 1971). Importantly, during experimental consumption not only the effects of a drug are explored at usually different doses and settings (Patrick and Maggs, 2008). At the same time it is also experimented with how the drug-effects on mental states can be “used” in relation to different settings (Zinberg, 1984; Simons et al., 2000). Thereby numerous episodic drug memories are formed which guide later parameters of consumption. Evidence for a systematic knowledge about drugs and drug-effects is provided by the elaborate expectancies that people develop toward the drug-effects before and during consumption (e.g., Brown et al., 1980; Brown, 1985; Gustafson, 1991; Peele and Brodsky, 2000). As such, an individual dose titration can be the consequence of a series of episodes with either too low or too high doses and perceived subjective and behavioral consequences.

The other important time when episodic memories guide future consumption is the establishment of drug instrumentalization. This occurs usually not after first consumption, but requires already some experience with the drug including the knowledge of an individual dose-effect relationship. The learning of drug-instrumentalization involves an extension of the episodic drug memory components in terms of the preconditions and consequences of behavior. However, it can still be a one-trial experience by which future drug use is shaped. Several reports from drug users show that the perceived and reported “usefulness” of the drug-effects was found to predict future use of the drugs (Boys et al., 1999; Boys and Marsden, 2003; Leigh and Stacy, 2004). For instance, a certain dose of alcohol can be a boring experience when consumed alone at home by a teenager, i.e., when no instrumentalization is possible, while the disinhibitory effects of the same dose may be found highly entertaining and rewarding at a party with peers and the opposite sex. In this case, an episodic drug memory is established which contributes to later drug instrumentalization. It can be retrieved at a future occasion to facilitate socializing and mating approaches.

It was argued that one pathway into addiction is by an attempted over-instrumentalization of drug-effects, e.g., under stressed conditions (Kippin, 2011; Müller and Schumann, 2011b). While episodic memories may contribute to it, important processes most likely involve classical and instrumental conditioning and habit learning (Everitt and Robbins, 2005). At present one cannot say if there is a role of episodic memories in the transition from regular drug use and instrumentalization to compulsive use and addiction.

## Acute Physiological Effects of Drugs in Memory Systems of the Brain

One way to investigate the neuronal mechanisms of episodic drug memories may be to look at the first encounter with an addictive drug. At this time, no conditioned drug-effects are established, and no adaptive or neurotoxic drug-effects have occurred in the brain. In this section I look at those acute drug-effects which might serve or influence episodic drug memories in drug naïve individuals.

### Dopamine and serotonin

Psychotropic drugs exert profound effects on extracellular neurotransmitter activity. A significant increase in dopaminergic (DA) activity was reported, which is particularly well documented in the mesolimbic system for alcohol (McBride et al., 2002; Tupala and Tiihonen, 2004; Spanagel, 2009), nicotine (Markou, 2008), cocaine (Di Chiara and Imperato, 1988; Johanson and Fischman, 1989; Müller et al., 2002), amphetamines (Seiden et al., 1993; Green et al., 2003; Müller et al., 2007a), opiates (Di Chiara and North, 1992; McBride et al., 1999), cannabis/marijuana (Ameri, 1999; Iversen, 2000), and caffeine (Cauli and Morelli, 2005). However, activation of DA activity was also described for, e.g., cocaine and amphetamine in the neocortex and the hippocampus and related cortical structures (Müller and Huston, 2007; Pum et al., 2007). There is now good evidence for a functional role of the DA effects in the mesolimbic system for the establishment of various addiction-related behaviors (Koob et al., 1998; Wise, 2002; Pierce and Kumaresan, 2006). The functional role in most memory-associated structures still has to be determined. Importantly, DA activation plays also an important functional role in various types of memory, such as working memory (Williams and Goldman-Rakic, 1995; Wang et al., 2004) and reward learning (Schultz, 2000; Willuhn et al., 2012). It may be speculated that a drug-induced DA activation is an early step in amplifying learning processes. Although DA is a central mechanism for addiction and learning and memory, it is by far not the only transmitter which is acutely activated. Various psychoactive drugs enhance also serotonergic activity (5-HT; Müller et al., 2004, 2007b, 2010). Animal studies showed that psychoactive drugs like cocaine and amphetamine induce strong 5-HT responses in several neocortical and hippocampus-associated regions (Müller et al., 2004, 2007b; Pum et al., 2007) which play important roles in components of the episodic memory (Aggleton and Brown, 1999; Dere et al., 2006, 2007).

Formation of an episodic memory depends on emotional valence of the perceived episode. The higher the emotional valence, the more likely it appears for the event to be remembered. High emotional impact is characterized by strong activation of emotion processing systems, such as serotonin responses in the amygdala for negative emotions (Rex et al., 1993; Pum et al., 2009) or dopamine responses in the nucleus accumbens for unexpected positive valence (Schultz, 2000; Willuhn et al., 2012). The neurochemical acute effects of psychoactive drugs in emotion processing brain areas usually exaggerate the activation by natural stimuli (Rueter et al., 1997; Müller et al., 2007a). This may result in a mock signal of emotional importance of the preceding events and behavior (Nesse and Berridge, 1997), but a very real episodic memory thereof.

### Acetylcholine

The acetylcholinergic (ACh) system plays an important role in attentional and memory processes (Blokland, 1995; Sarter et al., 2005) and is crucially involved in consciousness (Perry et al., 1999; Müller et al., 2011; Woolf and Butcher, 2011) and episodic memory (Dere et al., 2007). Acute application of cocaine was shown to activate cholinergic interneurons in the nucleus accumbens (Witten et al., 2010). Several drugs have been shown to increase extracellular ACh levels after acute application, such as cocaine, amphetamine, morphine, alcohol, and nicotine. It was observed in the ventral striatum, caudate nucleus, cortex, and ventral tegmental area (VTA), but most pronounced in the hippocampus (Imperato et al., 1992, 1993a, 1996; Quirion et al., 1994; Zocchi and Pert, 1994; Summers and Giacobini, 1995; Henn et al., 1998; Consolo et al., 1999; Larsson et al., 2005). The later effect was suggested to be a potential base for drug-effects on declarative memories (Williams and Adinoff, 2008). The nicotine-induced ACh increase is mediated by local nicotinergic ACh receptors (Summers and Giacobini, 1995; Tani et al., 1998). It was shown that the psychostimulant-induced ACh increase was not mediated by direct interaction with muscarinergic or nicotinergic ACh receptors, but depends mainly on DA-induced D1 receptor activation (Imperato et al., 1992, 1993a,b; Consolo et al., 1999). While a crucial role of the ACh system in addiction-related behaviors which require instrumental and classical conditioning is now evident (Bardo, 1998; McBride et al., 1999; Williams and Adinoff, 2008), a functional role of acute drug-effects in the ACh system for episodic drug memories still needs to be investigated.

### Glutamate

An important finding was that major drugs of addiction, such as cocaine, amphetamine, morphine, ethanol, and nicotine can enhance synaptic plasticity at excitatory synapses in VTA DA neurons, as measured by the ratio of alpha-amino-3-hydroxy-5-methyl-4-isoxazolepropionic acid receptor (AMPA)- and *N*-methyl-d-aspartate receptor (NMDA) dependent excitatory postsynaptic currents (EPSCs; Ungless et al., 2001; Saal et al., 2003; Faleiro et al., 2004). This plasticity is persistent for several days during which it may be the base for behavioral hypersensitivity (Borgland et al., 2004; Wanat and Bonci, 2008; Zweifel et al., 2008). Downstream mechanisms for this fast plasticity include protein synthesis, a reduced NMDA receptor function, and the active insertion of GluR2-lacking AMPA receptors, such as GluR1-containing AMPA receptors (Dong et al., 2004; Argilli et al., 2008; Mameli et al., 2011). GluR2-lacking AMPA receptors have a greater channel conductance and are Ca^2+^ permeable, which allows them to trigger also Ca^2+^ dependent signaling cascades (Bowers et al., 2010). It should be noted, however, that none of the investigated drugs induces addiction after a single administration in humans or animals. However, there is some learning involved already after a single drug exposure, which may in animals include a one-trial conditioned place preference (Zweifel et al., 2008), and also the memory of the self-administration episode. As such, early synaptic plasticity after acute drug exposure may be an important substrate of one-trial episodic drug memory. Neurophysiological studies have so far focused mainly on DA neurons in the VTA. Given the omnipresence of neuroplasticity at glutamatergic synapses in the brain and the dopamine-independent effects of some addictive drugs (Pierce and Kumaresan, 2006), it would be important to investigate the potential of addictive drugs to cause this early plasticity also in other memory-related synapses.

### Intracellular signaling cascades

The concept of drug memory proofed to be useful when animal studies revealed that many of the molecular changes at cellular level, which occurred after single and chronic drug administration, were similar to those observed during normal learning. Drugs of addiction, for example, acutely increased the levels the second messenger, cyclic adenosine monophosphate (cAMP), and activated the protein kinase A – cAMP-response element binding protein (CREB) cascade, which regulates gene transcription and protein synthesis. This leads, among others, to changes in the expression of NMDA and AMPA glutamate receptors (Nestler and Aghajanian, 1997; Hyman and Malenka, 2001; Nestler, 2002; Hyman et al., 2006; Kauer and Malenka, 2007). In particular CREB-regulated gene transcription and protein synthesis are believed to be essential mechanisms for long lasting cellular and behavioral plasticity (Kandel and Pittenger, 1999; Kandel, 2001). Another important pathway activated by drugs of addiction involves the second messenger Ca^2+^, calmodulin, and Ca^2+^/Calmodulin-dependent-kinases. Activation of this pathway is essential for various types of learning (Giese et al., 1998; Irvine et al., 2006; Easton et al., 2013b) as well as for several drug-related behaviors (Licata and Pierce, 2003; Liu et al., 2006; Anderson et al., 2008; Bilbao et al., 2008; Easton et al., 2013a). A series of functional studies showed that antisense oligodeoxynucleotides for the immediate early gene, Zif268, in the basolateral amygdala could disrupt different memories for cocaine and heroin (Lee et al., 2005, 2006; Hellemans et al., 2006). Altogether, drugs of addiction were shown to be very efficient in activating gene transcription factors (Nestler, 2008) and cellular plasticity after only a single drug exposure for several days (Hyman et al., 2006; Ungless et al., 2010). Both are indicators of cellular processes underlying fast learning (Morris et al., 1986; Aggleton and Brown, 2005).

### Neuronal morphology

There is a growing physiological base for drug-related memories in particular in structures of the brain’s reinforcement system (Kelley, 2004). However, the neurophysiological adaptations were not only observed in the reward system, but also in other structures of the brain. A series of studies in animals by Robinson and Kolb (Robinson and Kolb, 1997, 1999, 2004; Robinson et al., 2001; Li et al., 2003; Ferrario et al., 2005) showed that psychoactive drugs, such as cocaine, amphetamine, nicotine, and morphine can cause a number of morphological changes in neocortical neurons, i.e., in areas not considered to be part of the reward system, but rather part of other memory circuits (Eichenbaum, 1997; Milner et al., 1998). These changes, which comprise an increase in neuronal spine density and axonal sprouting, were normally observed during normal, i.e., drug free, learning (Moser et al., 1994). Furthermore, when induced by psychoactive drugs, normal environmental enrichment, was no longer able to induce it (Kolb et al., 2003).

### Neuroimaging

An activation of cortical memory areas was also shown in various imaging studies in humans during drug exposure or presentation of craving-eliciting cues (Grant et al., 1996; Bonson et al., 2002; Goldstein et al., 2007). Thereby, the brain areas activated during actual drug exposure often overlap with those areas activated during presentation of drug-associated cues (e.g., Breiter et al., 1997; Childress et al., 1999). These findings support the view that psychoactive drugs do not only functionally interact with the brain reward system, but also with other memory systems – if not with the whole brain (Tretter et al., 2009), thus causing acute responses and plastic changes which are similar to those observed during normal learning (Kelley, 2004).

While the review of neurophysiological drug-effects is far from complete, it shows several mechanisms of how an acute drug-induced activation of memory systems may result in fast plasticity which can shape future behavioral responses to drug-cues and the drug itself. How each of them relates specifically to episodic drug memories still has to be shown. It should also be noted, that these effects might reflect merely functional correlates of episodic drug memories during initial/early drug exposure. There are also consummatory episodes remembered during chronic consumption and addiction. However, after repeated drug exposition, many more drug memories are formed, which control future drug directed behaviors (Figure 1). Furthermore, episodic memories of a single consummatory episode may consist of rather different components, such as an initial euphoria episode later followed by an aversive withdrawal episode. Both are characterized by quite different neurochemical profiles (I have only discussed the initial effects here), but may result in equally well remembered episodes. Currently this may only suggest that quite different mechanisms may shape episodic drug memories, e.g., in their affective value, which clearly awaits further research.

Another limitation in the interpretation of neurophysiological drug-effects arises from a lack of proper models for episodic drug memories. Virtually all animal models of addiction, such as operant self-administration or conditioned place preference, focus on addiction-related behaviors which all require multiple drug expositions (Olmstead, 2006; Sanchis-Segura and Spanagel, 2006). Coinciding neuronal and functional adaptations in the brain may, thus, reflect more than one type of learning and memory (e.g., Huston et al., 2013). For future research in the episodic memories of drug-effects, a specific animal model would be useful.

## Conclusion

Here a potential role of episodic drug memories in psychoactive drug use and drug instrumentalization is suggested. Easily accessible verbal reports from human drug users suggest that there are episodic memories with specific drug-related content. These memories are, especially when involving strong emotional responses, one-trial memories with a particularly high extinction resistance. Neurophysiological analyses of drug-effects in the brain provide evidence for strong neurochemical effects of psychoactive drugs in memory processing systems, which outreach those of non-drug stimuli/events. As such, there emerges also a neurophysiological base for episodic drug memories which may influence later consummatory behaviors. A more in depth analysis of the concept suggests that the “What-Where-When” characteristic of episodic memories can be applied equally well to the analysis of episodic drug memories. Altogether, it is suggested to consider episodic drug memories as an important mediator in the establishment of early drug consumption and the transition to drug instrumentalization which should warrant further exploration.

## Conflict of Interest Statement

The authors declare that the research was conducted in the absence of any commercial or financial relationships that could be construed as a potential conflict of interest.
